# Neck circumference as a potential marker of metabolic syndrome among college students^1^

**DOI:** 10.1590/0104-1169.3565.2505

**Published:** 2014

**Authors:** Dayse Christina Rodrigues Pereira, Márcio Flávio Moura de Araújo, Roberto Wagner Júnior Freire de Freitas, Carla Regina de Souza Teixeira, Maria Lúcia Zanetti, Marta Maria Coelho Damasceno

**Affiliations:** 2 MSc, Professor, Faculdade de Juazeiro do Norte, Juazeiro do Norte, CE, Brazil; 3 PhD, Adjunct Professor, Instituto de Ciências da Saúde, Universidade da Integração Internacional da Lusofonia Afro Brasileira, Redenção, CE, Brasil; 4 PhD, Adjunct Professor, Departamento de Enfermagem, Universidade Federal do Piauí, Floriano, PI, Brazil; 5 PhD, Associate Professor, Escola de Enfermagem de Ribeirão Preto, Universidade de São Paulo, WHO Collaborating Centre for Nursing Research Development, Ribeirão Preto, SP, Brazil; 6 PhD, Adjunct Professor, Faculdade de Farmácia Odontologia e Enfermagem, Universidade Federal do Ceará, Fortaleza, CE, Brazil

**Keywords:** Anthropometry, Neck Circumference, Nursing, Diabetes Mellitus

## Abstract

**OBJECTIVE:**

to relate neck circumference with metabolic syndrome and its criteria among college students.

**METHOD:**

cross-sectional study conducted with 702 college students in Fortaleza, CE, Brazil from September 2010 to June 2011. Socio-demographic data, waist circumference and neck circumference were collected together with blood pressure, fasting blood sugar, triglyceride levels, and HDL-C.

**RESULTS:**

1.7% of the studied sample presented metabolic syndrome. Of these, 58.3% presented altered neck circumference (p<0.006). As neck circumference decreases, pressure levels improve (p<0.001). Additionally, college students with high fasting blood sugar (p=0.003) and high triglyceride levels (p<0.001) presented higher values of neck circumference.

**CONCLUSION:**

neck circumference is a potential predictive marker in the detection of metabolic syndrome and its components among college students.

## Introduction

The National Priorities for Research and the Research Agenda in the health field gives priority in one of its sub-agendas to non-communicable diseases (NCDs), such as hypertension, diabetes mellitus and obesity^(^ 1 ^)^. Metabolic syndrome (MS) is also added to this context, the diagnosis of which may include these diseases; the disease also increases the risk for developing cerebrovascular and coronary artery diseases^(^ 2 ^)^. Metabolic syndrome is defined as a separate entity based on risk factors for cardiovascular diseases and type-2 diabetes mellitus (DM2). Factors related to MS include visceral obesity, dyslipidemia, arterial hypertension, and insulin resistance. MS should be recognized before the clinical manifestation of DM2 in order to implement primary prevention through lifestyle modifications and should specifically treat each of the syndrome components^(^ 3 ^-^ 4 ^)^.

There are various sources providing criteria for identifying MS^(^ 3 ^-^ 4 ^)^, however, those established by the National Cholesterol Education Program - Adult Treatment Panel III (NCEP ATP III) and recommended by the I Brazilian Guideline for the Diagnosis and Treatment of Metabolic Syndrome, are more frequently used in clinical studies due to their practicality. These programs consider the presence of three or more criteria to indicate the presence of metabolic syndrome, namely: waist circumference greater than 88cm in women and 102 cm in men, triglycerides above 150mg/dl, HDL cholesterol below 40 mg/dl in men or 50mg/dl in women, blood pressure above or equal to 130/85 mmHg, and circulating glucose greater than or equal to 110 mg/dl^(^ 3 ^)^.

The search for easily applicable clinical criteria to identify MS led researchers to consider the importance of investigating other anthropometric parameters to integrate or replace some of those already established^(^ 5 ^)^. Therefore, neck circumference (NC) began to be investigated under the argument that there are limitations in regard to waist circumference measurement (i.e. lack of a standard measurement technique, variations in certain health conditions) and also because neck measurements, under normal conditions, do not oscillate throughout the day^(^ 6 ^-^ 8 ^)^. 

Hence, neck circumference is now considered a risk marker for MS, even though further investigation with different populations is still recommended^(^ 8 ^-^ 11 ^)^.

In Brazil, neck circumference was investigated as part of the Brazilian Metabolic Syndrome Study (BRAMS) that included adult outpatients (between 18 and 60 years old) undergoing treatment for DM2, metabolic syndrome and/or obesity. The results found so far show that neck circumference is an innovative and additional parameter to determine the distribution of body fat, which is associated with visceral fat, to metabolic syndrome's components and insulin resistance, especially among women^(^ 12 ^)^. 

The city of Fortaleza, CE, Brazil was included in BRAMS, but neck circumference as an MS marker has not yet been studied in predominantly young populations. Hence, considering anthropometry as a tool to be used in Nursing consultations and also that the National Priorities for Research and the Research Agenda in the health field point to the need to develop and use health-promoting methods in addition to preventive, diagnostic, and early treatment methods^(^ 1 ^)^, this study's objective was to relate neck circumference to metabolic syndrome and its criteria among college students. 

This study is justified by the scarcity of studies addressing this topic in Brazil and because it can support improved healthcare delivery, regardless of the context in which these actions are developed. 

## Method

This cross-sectional study was performed at the Federal University of Ceará (UFC) in the campuses of Fortaleza, CE, Brazil. The sample was calculated using a formula for infinite populations. A prevalence of 50% was adopted because it provides the maximum sample size, with a level of significance of α=0.05 and an absolute sampling error of 4%. To minimize potential losses, 10% was added to the sample size (n=702 college students). The participants were distributed in the following fields: human sciences, exact sciences, agricultural sciences, health sciences, sciences and technology. Based on the population per field of knowledge, the sample was stratified in such a way that 143, 116, 98, 106, 127 and 112 students were studied, respectively.

Students from the following programs participated in the study: Pedagogy, Social Sciences and Letters in the area of the human sciences; Business, Economy and Accounting Programs from the exact sciences; Animal Science, Agronomy, and Fishing Engineering from the agricultural sciences; from the Nursing and Pharmacy programs from the health sciences; and Chemistry, Geography from the sciences; biological sciences and technology; and Electrical, Metallurgical, and Civil Engineering. 

Inclusion criteria were: being properly enrolled in brick-and-mortar undergraduate programs in daytime hours; living in Fortaleza, CE, Brazil; and having a phone number and email. Pregnancy was an exclusion criterion.

Socio demographic variables included: sex (male and female); age (16-58 years old); race (Caucasian, biracial, Afro-descendant, Asian); marital status (married/stable union, single, widowed, separated/divorced); occupational situation (whether only study or also work); grade (current semester); beginner (is attending first half of the program); senior (is attending second half of the program); economic situation (A1, A2, B1, B2, C, D, E according to the Criteria of Brazil Economic Classification established by the Brazilian Association of Business and Research)^(^ 13 ^)^, and with whom the individual lives with (parents, relatives, friends, spouse/partner, alone).

The following were established for anthropometric, clinical and biochemical variables: central obesity (waist circumference [WC] greater than 102cm for men and 88cm for women); neck circumference [NC] greater than or equal to 39cm for men and greater than or equal to 35cm for women)^(^ 12 ^)^; high blood pressure (greater or equal to 130/85 mmHg); high triglycerides level (greater or equal to 150 mg/dl); low HDL cholesterol level (below 40mg/dl for men and below 50mg/dl for women); and circulating glucose greater than or equal to 110mg/dl)^(^ 3 ^)^.

The researcher assistants (two doctoral and two master's students along with two undergraduate students) responsible for collecting data received 30 hours of training to ensure the reliability of data. Data collection was performed from September to November 2010 and from February to June 2011 in private rooms on the university's premise.

The students were recruited in classrooms after the study's objectives and methodology was clarified. Those who consented signed free and informed consent forms and completed a questionnaire addressing socio-demographic data. The day and time for measuring neck circumference (NC), waist circumference (WC) and blood pressure (BP), and collecting blood samples, was also scheduled. The researchers contacted the students on the day before the scheduled time to remind them of the need to fast for 12 hours. 

The participants stood erect with their heads positioned in the Frankfurt horizontal plane at the time of measuring NC. An inelastic metric tape was placed at the midpoint of the neck's height. The measure was taken immediately below Adam's apple^(14-16) ^among the male participants. Waist circumference (WC) was measured with an inelastic tape placed on the skin at the midpoint between the iliac crest and the last rib at the end of the expiratory breathing^(^ 16 ^-^ 17 ^)^. Blood pressure was measured in accordance with Brazilian guidelines^(^ 18 ^)^. The collection of a venous blood sample was performed by technicians from a specialized laboratory in accordance with standards guiding the preservation of samples and the safety of individuals. A free snack was provided after blood sample collection. The project was approved by the Institutional Review Board guiding research with human subjects at the Federal University of Ceará (Protocol No. 208/10).

Data were triple-entered into Excel and then imported into IBM-SPSS, version 14.0. Statistical measures, average, standard deviation, and odds ratios were computed with their respective confidence intervals of 95% (CI_95%_). Before comparing averages, we verified the normality of data and equality of variances using the Kolmogorov-Smirnov and Levene tests, respectively. NC averages were analyzed using Student's t-test for independent data and Snedecor's F-test. In the latter, when p<0.05, multiple comparisons were performed using the Tukey test (if variations were equal) or the Games-Howell test (if variances were not equal). Associations among NC and risk groups were analyzed using the Chi-square (χ^2)^.

## Results

Of the 702 college students, 62.7% were women; 53.3% were aged between 20 and 24 years old (21.5±1.57); 49.3% were biracial; 92.3% were single; 69.1% were in their first to the fifth semester; 71.2 % lived with their parents; and 65.2 % studied only. There was a predominance of socioeconomic classes B and C (39.5%), with monthly family income of U$ 1,705 dollars (SD=200). This variable presented an asymmetric distribution at right (Kolmogorov Sminorv com p<0.001).

When comparing the averages of association between NC and the socio-demographic data, statistically significant association was found in regard to sex (p<0.001), age (p=0.009), and occupational situation (p<0.001). Therefore, male students aged ≥ 25 years old and those who worked besides studying presented the highest measure of neck circumference.

The NC of men and women who presented high WC was greater than that of individuals with normal NC (p<0.001). Additionally, as NC decreases, the BP of the participants improves (p<0.001). Individuals with high FBS (p=0.003) and TG (p<0.001) presented higher NC, as opposed to what happened with HDL-C (Table 1).

**Table 1 -:** Association between Neck Circumference and the components of Metabolic Syndrome. Fortaleza, CE, Brazil, 2011

Variables		High			Under normal limits		RC: CI_95%_	P value
		n	%		n	%		
Waist circumference (WC) under normal limits								
	Male	128	18.8		554	81.2	1.7; 1.2-2.5	<0.001
	Female	68	11.4		528	88.6	1	
High waist circumference (WC)								
	Male	18	100		-	-	1.3; 1.1-1.4	<0.017
	Female	78	75.0		26	25.0	1	
Blood pressure								
	Hypertension	12	55.0		9	45.0	7.5; 2.8 – 20.8	<0.001
	Borderline	14	45.2		17	54.8	4.6; 2.0 -10.4	<0.001
	Normal	36	40.4		53	59.6	3.8; 2.2 -6.3	<0.001
	Excellent	84	15.0		475	85.0	1	
Fasting blood sugar (FBS)								
	Under normal parameters	28	33.3		56	66.7	2.0; 1.2 – 3.5	<0.005
	Above normal parameters	117	19.3		489	80.7	1	
Triglycerides (TG)								
	Under normal parameters	51	32.1		108	67.9	2.1; 1.4 – 3.3	<0.001
	Above normal parameters	94	17.7		436	82.3	1	
High density lipoprotein (HDL-C)								
	Under normal parameters	8	9.8		75	90.2	0.3; 0.1 – 0.7	<0.004
	Above normal parameters	137	22.6		469	77.4	1	

The percentages of students with changes simultaneously between NC and the biochemical criteria of MS, that is, FBS (p< 0.005), TG (p< 0.001) and HDL-C (p<0.004), were 33.3%, 32.1% and 9.8%, respectively. These values were lower among those with normal biochemical measures and NC (Table 1).

Only 1.7% of the sample present MS, 58.3% of which simultaneously presented MS and altered NC (p< 0.006). Additionally, individuals with MS presented higher NC in regard to the other participants (p<0.001) (Table 3).

**Table 2 -:** Comparison of averages concerning association between Neck Circumference and the components of Metabolic Syndrome. Fortaleza, CE, Brazil, 2011

Variables			Average	± Standard error of the mean	P value
Waist circumference (WC)					
	Male				<0.001
		Under normal parameters	33.61	0.123	
		Above normal parameters	41.06	0.631	
	Female				<0.001
		Under normal parameters	33.00	0.115	
		Above normal parameters	38.37	0.279	
Blood pressure					<0.001
		Excellent	33.12	0.130	
		Normal	36.00	0.358	
		Borderline	37.53	0.448	
		Hypertension	37.13	0.854	
Fasting blood sugar (FBS)					0.003
		Under normal parameters	33.67	0.136	
		Above normal parameters	34.84	0.396	
Triglycerides					<0.001
		Under normal parameters	33.53	0.139	
		Above normal parameters	34.79	0.306	
High density lipoprotein (HDL-C)					<0.001
		Under normal parameters	33.99	0.141	
		Above normal parameters	32.57	0.285	

**Table 3 -:** Association between Neck Circumference and Metabolic Syndrome among college students. Fortaleza, CE, Brazil, 2011

Variables		Above normal parameters			Under normal parameters		RC: CI_95%_	P value	Mean	Standard error of the mean	P value
		n	%		n	%					
Metabolic Syndrome								< 0.006			< 0.001
	Yes	7	58.3		5	41.7	5.4; 1.4-22.1		37.04	1.199	
	No	138	20.4		539	79.6	1		33.76	0.129	

## Discussion

The relationship between NC and sex showed that men presented higher neck circumference values. Other studies, however, found this association in both sexes and this issue has not been totally clarified^(^ 9 ^,^ 12 ^,^ 14 ^)^. It is possible that the predominance of excess weight among women explains the greater female vulnerability to higher NC^(^ 19 ^)^.

Despite the prevalence of MS being low among college students, a significant association was found with high NC. Additionally, those with one or two MS components, also presented a higher average NC; however, more robust evidence was detected in regard to NC and WC, NC and BP, and NC and TG. In this sense, this study's results are in agreement with those found in recent literature^(^ 20 ^-^ 23 ^)^.

Hence, the measurement of NC may help to diagnose MS and various health problems, such as central obesity, pre-hypertension, hypertension and dyslipidemia, in addition to indicating the condition of being overweight and obese^(^ 24 ^)^. One study addressing the relationship between NC and relative muscle force and cardiovascular risk factors among sedentary women showed that NC predicts cardiovascular risk^(^ 25 ^)^. On the other hand, another study with nursing professionals indicates that NC is not sensitive enough to capture metabolic deviations related to blood lipoprotein, as in other measures of body composition^(^ 26 ^)^.

The measurement of NC is a simple, reliable and low cost procedure that enables it to be implemented in primary care by any health worker, whether to prevent diseases or to identify them, reaching large and different populations. 

 Because this is a cross-sectional study, however, we cannot draw causal relationships, not even for the MS components with statistically significant associations. The criteria used to identify MS may underestimate the number of cases, as they consider the difference of values among criteria established by organizations in regard to central obesity^(^ 27 ^)^.

A lack of Brazilian studies addressing this topic, regardless of the segment of population, as well as of international investigations involving college students, hindered a deeper discussion and comparisons with literature, though it may contribute to BRAMS.

At the same time, this study points to the need to develop further studies including various and different populations in terms of ethnicity, age, health conditions, and other criteria for the diagnosis of MS, to enable comparisons and better clarify NC as an MS marker. Additionally, this study guides those interested in the topic to review current, established criteria to classify MS and avoid underestimation of cases, as well as to consider the possibility of including neck circumference as a marker for metabolic syndrome.

## Conclusions

Neck circumference was associated with MS and all its criteria according to NCEP/ATP III. Therefore, NC is a potential marker as an additional parameter to screen for MS among college students. 
